# Editorial to accompany the special issue: Social and health inequities in chronic pain across the life span

**DOI:** 10.1080/24740527.2025.2533301

**Published:** 2025-08-21

**Authors:** Fiona Webster, Leigha Comer, Abhimanyu Sud, Kara Turcotte, Joel Katz

**Affiliations:** Arthur and Sonia Labatt Family School of Nursing, Faculty of Health Sciences, Western University, London, ON, Canada; School of Health Studies, Faculty of Health Sciences, Western University, London, ON, Canada; Department of Family and Community Medicine, Temerty Faculty of Medicine, Toronto, ON, Canada; Humber River Health, Toronto, ON, Canada; Faculty of Applied Science, The University of British Columbia School of Nursing, Vancouver, BC, Canada; Department of Psychology, York University, Toronto, ON, Canada; Department of Anesthesia and Pain Management, Toronto General Hospital, Toronto, ON, Canada; Department of Anesthesiology and Pain Medicine, Temerty Faculty of Medicine, University of Toronto, Toronto, ON, Canada

## Introduction

The special issue on social and health inequities in chronic pain across the life span reflects the growing awareness of the social and health inequities that characterize many people’s experiences of chronic pain. Though we know that people living with chronic pain often face stigma, discrimination, and multiple barriers to accessing health care, and though chronic pain is overrepresented among marginalized groups, few studies focus specifically on drivers of marginalization, such as racism, colonialism, stigma, ableism, exclusion, marginalization, poverty, and food insecurity. It seemed both timely and urgent to bring together in one issue research that was attentive to these sources of profound inequities, especially in a historical moment when many rights of marginalized communities are under threat.

Affecting one in four Canadians over the age of 15,^1^ chronic pain is a stigmatized health condition due, in part, to its subjective nature,^2,3^ as evidenced by inadequate funding for health services^1^ and research.^4^ Chronic pain is further marked by “prestige rankings” whereby certain conditions, like fibromyalgia, are dismissed or invalidated more easily than others depending on the characteristics of those who experience these conditions due to intersecting structures of power, such as gender bias^5^ and racism,^6^ resulting in personal devaluation and internalized stigma among those living with pain.^7^ There is also growing recognition of the association between pain and systemic and structural marginalization,^1,8–10^ inclusive of lifetime experiences of trauma and violence.^11,12^

In response to our call for papers, we received submissions from authors working in Canada and the United States across a wide range of disciplines, including epidemiology, Indigenous health, kinesiology, medicine, nursing, psychology, public health, rehabilitation science, science and technology studies, and sociology. The articles included in this issue reflect diverse methodological approaches, ranging from descriptive and critical qualitative research to scoping reviews, needs assessments, and mixed methods and quantitative studies. What all of the manuscripts hold in common, however, is a strong interest in issues of social inequities and a commitment to social justice.

What follows is a brief overview of each article included in the special issue. Though the papers vary in population, methodology, and disciplinary lens, they share a commitment to examining how pain is shaped by broader social and structural forces. These summaries offer a window into the innovative, equity-informed work being carried out across the field and set the stage for deeper reflection on the shared insights, tensions, and questions that emerge when chronic pain is studied through the lens of justice.

## Overview of contributions

The team led by Connoy^13^ undertook a scoping review focused on chronic pain and marginalization that drew on 67 studies. Moving beyond description, they offer a critical review that makes visible several important themes. They highlight the varying degrees of consideration of sociopolitical and socioeconomic contexts within the literature, the conceptual conflations between sex and gender, and the differing approaches to how people living with chronic pain and marginalization are described. Their review identifies the need for greater conceptual clarity and application of the notion of marginalization within the field, the importance of paying greater attention to explicating processes of marginalization, and the need to attend to unconscious biases rendered evident through researcher language and generalizing references to heterogeneous populations.

In their provocatively but aptly titled article, Crocket et al.^14^ offer an analysis from the first stage of a multistage project in which they explore the care-seeking experiences of people living with chronic back pain in Saskatchewan and specifically the unique experiences facing urban, rural, remote, and/or Indigenous peoples. In their analysis, the authors highlight challenges to accessing care, which include travel as a main barrier, as well as facilitators to access, such as funded care and care closer to home. They also explore participant recommendations for improved care provision, such as coordination of care and patient-centered care, and the importance of communication with health care providers.

Ul Haq et al.^15^ explore the experiences of Canadian Armed Forces veterans living with chronic pain, their transition from military to civilian care, perceived barriers and facilitators to chronic pain care, and the impacts of pain on domains of well-being. Through a descriptive qualitative study engaging 35 veterans with chronic pain, the authors nestle veterans’ accounts of pain within the unique features of military life, including a mission-centric culture and an obligation to fulfil duties, which frame pain as something to be pushed through. The authors also identify gendered aspects of military culture, such as ideas about masculinity that discourage people from seeking care. Based on their findings, they underline the importance of attending to the unique challenges faced by women and racialized people in the armed forces, as well as the value of a holistic approach that considers veterans’ health, activities, finances, social integration, skills, and physical, cultural, and social environments.

Mustafa et al.^16^ utilized an arts-based method to explore immigrant Indian women’s chronic pain experiences in Canada and enhance the understanding of those experiences by creating a visual opportunity for them to share their stories. The women’s photographs, as well as their descriptions of these photographs, provide a vivid entry into their daily lives. The authors analyze these photographs and texts through themes of bodies in pain, transversing spaces, and the methods women adopt to manage their pain. One of the authors’ most fascinating insights is the clash between culturally shaped gender roles and changing gender norms throughout processes of immigration, which shape the women’s representations of pain.

Zhang et al.^17^ conducted a needs assessment in collaboration with local health care providers as well as community members to gain insight into their community’s priorities, strengths, and concerns regarding chronic musculoskeletal pain management. Five recommendations were developed, and community engagement and relationship building were identified as essential to ensuring that cultural protocols were respected and community worldviews were accurately represented. The authors also discuss the obstacles faced by community members seeking equitable care, including systemic oppression, barriers to communication, and a sense of being unheard by health care providers.

Charette and Schaffzin^18^ present historical and contemporary case studies of pain measurement through theoretical frames that explicate the social construction of pain as a phenomenon whose quantification has been justified with statistical approaches that are often at odds with personal experience. They offer a fascinating and provocative history of measurement from a science and technology studies lens that throws into relief the ongoing tensions between the subjective nature of pain and the measures used to try to render it in objectivist terms. The authors also consider existing alternatives, as well as the actual and potential consequences of persisting with basing care based on quantified, data-based pain ratings.

Koscielniak et al.^19^ used storytelling, centered around the metaphor of weaving, to discuss the conception and implementation of the Project Extension for Community Healthcare Outcomes for Indigenous Chronic Pain and Substance Use Health following guidance from the project Elder. Grounded in storytelling as a form of knowledge gathering and dissemination, the paper engages storytelling as a means of speaking directly to how people come to know and make meaning out of their experiences and the world. The authors also draw on Two-Eyed Seeing as an approach that encourages equal consideration of Indigenous and Western knowledge systems. In their paper, they share the story of how they developed the project, including processes of weaving together knowledge systems and reconstituting deficit-based narratives.

Tracy et al.^20^ explore differences in psychosocial variables between transgender and gender-diverse and cisgender youth with chronic pain. Drawing on the minority stress model, their study highlights how intersecting forms of marginalization may shape psychosocial outcomes in ways that challenge clinical expectations. Their findings invite deeper reflection on the role of gender-affirming, equity-oriented care in improving quality of life for transgender and gender-diverse youth living with pain, as well as the need for further research on these experiences.

Mulchan et al.^21^ evaluate a novel implicit social and racial bias reduction intervention for providers treating youth with sickle cell disease and leukemia. Using virtual patient simulations and Zoom-based training in individuation and perspective taking, the study explores how implicit bias might be disrupted in clinical pain assessment. Though feasibility concerns emerged, participants found the intervention relevant and applicable, pointing to its potential for shaping more equitable provider decision making. The work speaks directly to the urgent need to confront racism in pain care, particularly for Black youth, whose pain is too often dismissed or undertreated.

Limani and Zajacova^22^ used cross-sectional survey data from the NEST Omnibus survey to identify significant differences in the prevalence of frequent pain and interfering pain across racial and ethnic groups in Canada using operationalizations of race and ethnicity following Statistics Canada definitions and recommendations. The researchers calculated the prevalence of pain among White, Black, East/Southeast Asian, South Asian, Indigenous, Multiracial, and “other” groups. Their findings highlight substantial racial/ethnic disparities in pain prevalence among Canadian adults. Given the historical inattention to race and ethnicity in the Canadian health research ecosystem, this study provides important and rigorous basis for further study.

## Shared themes and reflections

The contributions to this special issue collectively offer a rich, multidimensional picture of chronic pain as a phenomenon deeply embedded in social context. Though they span a wide range of methods, populations, and analytic frames, several shared commitments and recurring themes emerge.

A common thread across many papers is a commitment to centering lived experience. Whether through interviews with veterans, arts-based collaborations with immigrant women, or participatory assessments grounded in Indigenous knowledge systems, these studies prioritize the voices and expertise of those living with pain. This centering not only grounds the research in context but also challenges dominant narratives that can pathologize or depoliticize pain. Several papers also engage with methodological creativity as a form of justice-seeking. This methodological plurality points to a shared desire to expand ways of knowing in ways that are responsive to people’s everyday lives and the many dimensions of pain.

Another shared feature is the interrogation of invisibility, including efforts to render visible experiences, needs, and structural conditions that are often overlooked in pain research. This special issue brings attention to groups frequently excluded or underrepresented in pain studies, such as transgender and gender-diverse youth, racialized communities, Indigenous people, immigrants, and veterans, among others. These papers underscore how marginalization operates not only through limited access to care but also through epistemic exclusion, where certain kinds of knowledge and ways of knowing are dismissed or ignored.

Across the papers, authors also grapple with questions of equity in clinical care. They surface critical questions and tensions, including how implicit bias shapes assessment and treatment, how systemic barriers limit access, and how pain is read differently depending on the social location of the person expressing it. These papers not only highlight the barriers people face but also offer examples of more just, responsive, and culturally rooted approaches to care.

Importantly, the papers do not always arrive at neat conclusions. In fact, many raise generative tensions between individual and systemic framings of pain, the desire for generalizability and the need for specificity, and between the clinical imperative to measure and the lived reality of ambiguity. These tensions are not shortcomings but rather invitations to continue grappling with the complexity of pain in context.

Together, these papers push the field of pain research to consider not just who experiences pain but how pain is shaped by systems of power and what kinds of knowledge are made possible when we center equity, context, and lived experience.

## Conclusion

We hope that this special issue offers a useful introduction to the diversity and importance of defining and calling attention to social and health inequities in chronic pain across the lifespan. Taken together, the articles in this special issue highlight the importance of attending to the social and health inequities that shape many people’s experiences of chronic pain. Each contribution offers a distinct lens on how pain is lived, understood, and addressed within diverse social, cultural, and structural contexts. From arts-based research with immigrant women to quantitative analyses of racial disparities, from needs assessments rooted in community engagement to theoretical reflections on the meaning and measurement of pain, these papers collectively broaden the conversation. The work assembled here also underscores the value of interdisciplinary, equity-informed inquiry and points to the need for continued attention to the social dimensions of chronic pain. By exploring pain as not only a clinical concern but also a social and structural one, this issue contributes to a growing body of knowledge that seeks to improve care, understanding, and quality of life for people living with pain.
